# Realistic Performance Estimation in Small-Cohort Deep Learning and Machine Learning: A Leakage-Controlled Codebase Applied to Meningioma Response Prediction After Gamma Knife Radiosurgery

**DOI:** 10.3390/cancers18213590

**Published:** 2026-11-09

**Authors:** Herwin Speckter, Marko Radulovic, Ivan Gonzalez, Giancarlo Hernandez, Jose Bido, Diones Rivera, Luis Suazo, Santiago Valenzuela, Ismael Peralta, Jeffrey Paulino, Teuddis Bernard, Issael Ramirez, Peter Stoeter, Velicko Vranes

**Affiliations:** 1 Centro Gamma Knife Dominicano, Centro de Diagnóstico y Medicina Avanzada y de Conferencias Médicas y Telemedicina, Plaza de la Salud, Santo Domingo 10514, Dominican Republicivanzakigonzalez1@gmail.com (I.G.); neurogh@hotmail.com (G.H.); josebido1@yahoo.com (J.B.); dionesrivera@gmail.com (D.R.); lsuazo@cedimat.net (L.S.); stgo.sosa@gmail.com (S.V.); iperaltabaez@gmail.com (I.P.); jepaulino@cedimat.net (J.P.); tj.bernard@gmail.com (T.B.); issaelramirez@gmail.com (I.R.); peter.stoeter@gmx.de (P.S.); 2 Department of Radiology, CEDIMAT, Plaza de la Salud, Santo Domingo 10511, Dominican Republic; 3 Department of Basic and Environmental Science, Instituto Tecnológico de Santo Domingo (INTEC), Santo Domingo 10602, Dominican Republic; velicko.vranes@intec.edu.do; 4 Department of Experimental Oncology, Institute for Oncology & Radiology of Serbia, 11000 Belgrade, Serbia

**Keywords:** deep learning, machine learning, meningioma, Gamma Knife radiosurgery, therapy response

## Abstract

**Background/Objectives:** Deep learning may support the prediction of meningioma response after Gamma Knife radiosurgery (GKRS), but small clinical cohorts increase the risk of overfitting and unstable performance. We developed leakage-controlled code adjusted for small clinical datasets. **Methods:** Pretreatment contrast-enhanced T1 MRI from 190 patients was analysed using one tumour-centred axial slice per patient. Response was defined as a tumour volume decrease ≥ 30% per log_10_ (months). ResNet-10t, ResNet-18, ResNet-50 and DenseNet-121 with ImageNet or RadImageNet pretraining were evaluated in three workflows: (1) frozen backbone CNN, head-only training; (2) logistic regression or naive Bayes classification of features extracted from frozen CNNs; and (3) restricted CNN fine-tuning. Five partially overlapping chronological development/test splits were assessed, with all data-dependent fitting, feature selection and model optimisation restricted to development data. **Results:** The smallest RadImageNet ResNet-10t CNN performed best when fine-tuned (peak AUCs 0.953 and 1.000) but showed the widest split-to-split variation (range 0.738–1.000). When RadImageNet features extracted from ResNet-10t were classified by naive Bayes and logistic regression, they reached lower median and peak AUCs but within narrower ranges. Predictive performance did not consistently depend on CNN size. **Conclusions:** To our knowledge, this is the first deep learning study predicting volumetric meningioma response after GKRS. We provide openly available, ready-to-use code for three major workflows in small clinical datasets, with safeguards against information leakage. The benefit of fine-tuning and of RadImageNet pretraining depended on architecture. Robust small-cohort modelling requires attention to performance consistency across chronological splits, not only to the highest AUC in a single split.

## Simple Summary

This study investigated whether artificial intelligence could predict Gamma Knife radiosurgery (GKRS) response using pretreatment MRI scans from a cohort of 190 patients. Because small datasets are prone to overfitting and overly optimistic results, we implemented strict methodological safeguards to prevent data leakage during training. To assess the robustness of model performance, we evaluated each model across five chronological splits of the cohort. While certain deep learning models achieved high predictive accuracy, their performance varied substantially across the chronological splits. By contrast, traditional machine learning approaches yielded lower peak accuracy but maintained greater consistency across splits. As part of this study, we provide comprehensive, ready-to-use code covering three major deep learning and machine learning workflows optimised for small clinical datasets. Overall, AI models applied to pretreatment MRI showed promising ability to predict GKRS response, but no single modelling strategy was uniformly superior, which highlights the need to assess both predictive performance and stability across multiple cohort partitions.

## 1. Introduction

Deep learning is a powerful approach for extracting clinically meaningful information from medical images because it learns complex morphological and textural features directly from image data rather than relying on predefined radiomic features [1]. However, across 69 studies that compared deep learning models with models based on handcrafted radiomic features, the median AUC advantage of deep models was only 0.045 on internal validation and 0.025 on external validation at a median training size of 196 patients. This is probably because the performance of deep learning typically depends on large patient cohorts, which are often difficult to assemble in clinical research.

Approaches specifically adapted to smaller cohorts are therefore needed, as convolutional neural network (CNN) training in such settings is particularly prone to overfitting and poor generalisation. Architectures with fewer trainable parameters are generally more appropriate for small datasets [1], and the influence of network structure increases as cohort size decreases [2]. Regularisation such as dropout, weight decay and early stopping can further reduce overfitting [3]. Data augmentation can additionally improve training-set variability through transformed or synthetic images, ranging from simple geometric and intensity transformations to generative approaches [4,5]. In 3D imaging, analysing representative 2D slices reduces model complexity while retaining the in-plane tumour morphology on which many imaging predictors depend [6]. How much of a pretrained network should be fine-tuned on the target cohort is not fixed but rather depends on cohort size and on similarity to the pretraining data [7]. Small cohorts also limit what can be concluded from any single evaluation, because performance estimates derived from a small heldout test set vary widely between splits, and conventional cross-validation reporting tends to understate that variability [8,9].

We addressed the combined problem of overfitting and unstable evaluation in small cohorts in a clinically relevant task: the prediction of Gamma Knife radiosurgery (GKRS) response in meningioma from pretreatment MRI. GKRS is an established treatment for selected intracranial meningiomas because of its precision and efficacy, but tumour response varies substantially between patients. A reliable MRI-based model capable of predicting the degree of response before treatment could therefore support treatment personalisation and follow-up planning. Previous work on this endpoint in meningioma has relied on handcrafted radiomics features. Texture analysis identified intratumoural signal heterogeneity as an important predictor of GKRS response [10], and a pretreatment radiomic score achieved an AUC of 0.81 in 93 patients, which rose to 0.88 after the addition of Karnofsky performance status, although without a nested workflow [11]. A subsequent fully nested consensus machine learning analysis in 204 patients achieved test-set AUCs of 0.77 to 0.84 [12]. Whether the prediction of GKRS response in meningioma could be improved by the use of deep learning has not been established.

Radiosurgery response prediction has been approached using deep learning in other intracranial lesions. In brain metastases, a CNN reached a mean AUC of 0.878 in 194 patients, although this depended on posttreatment imaging and fell to 0.672 using the pretreatment scan alone [13]. Across nine machine learning studies of vestibular schwannoma after radiosurgery, comprising 1095 patients, the pooled AUC was 0.845, but classical machine learning was mostly used rather than deep learning [14]. The closest precedent to our current study compared training from scratch, transfer learning and fine-tuning for predicting the early response of brain metastases to Gamma Knife radiosurgery, but it randomly assigned 3194 images from 19 patients to training, validation and test sets such that slices from the same tumour appeared in more than one partition [15]. The random assignment of images rather than patients constitutes information leakage, because adjacent slices of the same tumour are near-duplicates. The model is therefore evaluated on tumours it has already seen, rather than on unseen ones.

The present study compares smaller and larger CNN backbones and evaluates domain-specific RadImageNet weights pretrained on approximately 1.35 million radiological images [16] against general-purpose transfer learning using ImageNet weights pretrained on approximately 1.28 million natural images [17]. Three complementary strategies were examined: end-to-end classification with restricted fine-tuning, end-to-end classification with a frozen CNN in which only the final classification layer was trained, and fixed CNN-derived features classified by lower-complexity machine learning models such as logistic regression and Gaussian naive Bayes.

A further major aspect of this study was the use of chronologically ordered development and test partitions, with earlier patients used for model development and later patients reserved as a holdout cohort. This reproduces the way a model is actually used within an institution, where each newly presenting patient is predicted from a cohort treated earlier. Random splitting evaluates a scenario that cannot arise in practice.

The main methodological novelties also include the complete executable code for implementing the full analysis pipeline, written so that it can be applied by researchers without programming experience. Existing open software addresses model construction rather than evaluation design. MONAI supplies components that the user must still assemble into a complete study [18], nnU-Net is confined to segmentation [19] and commercial code-free platforms are black boxes that do not publish their partitioning or model selection procedures [20,21]. Accordingly, information leakage remains common, affecting a very large proportion of published studies [22]. To our knowledge, no openly available pipeline for small clinical imaging cohorts combines strict leakage control and chronological holdout evaluation with the systematic comparison of pretraining sources, CNN architectures, fine-tuning strategies and downstream classifiers. The pipeline runs directly on hosted cloud hardware, requires no local installation and no command-line use, and is configured by editing a single block of settings at the top of the file so that a researcher only needs to supply the path to their own images and masks.

The aim of this study was to develop and rigorously evaluate deep learning strategies for predicting GKRS response in a small meningioma cohort, systematically examining four factors that affect the performance under limited-data conditions: the value of restricted CNN fine-tuning, the effect of CNN backbone size, ImageNet versus RadImageNet CNN pretraining, and whether CNN-derived features are classified more reliably by lower-complexity machine learning models. A further aim was to assess how prediction performance is affected by the development/test partition, by repeating the partitions across five chronological cutoffs in which earlier patients were used for development while later patients formed the holdout test cohort.

## 2. Materials and Methods

The present study was reported in accordance with the STROBE statement for cohort studies and with TRIPOD+AI [23], as incomplete reporting of architecture, hyperparameters and validation has been identified as a major weakness of small-data imaging studies [1].

### 2.1. Ethics Approval Statement

This study was approved by the CEDIMAT Institutional Review Board (CEI-290) and conducted in accordance with the Declaration of Helsinki and the World Medical Association’s Code of Ethics, first published in 1964 and last revised in 2013. Because the analysis was retrospective, the ethics committee waived the requirement for written informed consent.

### 2.2. Patients

The cohort comprised 190 patients with histologically confirmed or radiologically presumed meningioma, all treated at our Gamma Knife centre. Ages at treatment spanned 15.2 to 88.7 years (mean 53.5). Magnetic resonance imaging (MRI) was obtained within 4 weeks before radiosurgery, and every patient had at least 12 months of imaging follow-up (mean 47.5 months, range 12 to 152 months; Table 1). Mean tumour volume was 8.3 cm^3^ (range 0.3 to 50.9 cm^3^).

Tumours were located at the skull base in 124 cases, the convexity in 57 cases, the falx in 4 cases, the tentorium in 3 cases and intraventricularly in 2 cases. Prior surgery had been performed in 82 patients, with histology available for 23 of them: 17 meningothelial or psammomatous, 5 fibroblastic and 1 transitional meningioma, none with atypical features. In the remaining patients, WHO grade I was assumed from typical radiological features, namely, homogeneous contrast enhancement, a dural tail, no extension through cranial foramina, little or no peritumoural oedema and the absence of marked lobulation. Treatment based on an imaging diagnosis without histological confirmation is an established practice in appropriately selected patients undergoing SRS for presumed benign meningiomas [24].

**Table 1:** Patients, treatment characteristics and treatment results.

Patient and Treatment Characteristics	Value	Range
Number of patients	190	-
Age in years (mean, range)	53.5	(15.2/88.7)
Pre-SRS tumour volume in cm^3^ (mean, range)	8.3	(0.3/50.9)
Previous RT, SRS	0 [0%]	-
Previous surgeries	82 [43.2%]	-
Single-fraction SRS treatments	165	-
Hypofractionated SRS treatments	25	-
Number of fractions (mean, range)	1.34	(1/4)
Coverage index (mean, range)	96.3%	(67.0%/100%)
Selectivity index (mean, range)	70.7%	(16.0%/93.0%)
Paddick conformity index (mean, range [25])	68.0%	(16.0%/92.1%)
Margin physical dose in Gy (mean, range)	14.6	(11.0/24.0)
Maximum physical dose in Gy (mean, range)	25.5	(8.1/36.2)
Margin BED in Gy (mean, range)	62.8	(33.0/104.2)
Margin SFED in Gy (mean, range)	13.5	(9.4/18.0)
**Treatment results**	**Value**	**Range**
Follow-up period in months (mean, range)	47.5	(12.0/152.0)
Complete response (CR)	0 [0%]	-
Partial response (PR, decrease by ≥50%)	40 [21.1%]	-
Minor response (MR, ≥25% and <50% decrease)	77 [40.5%]	-
Stable disease (SD, <25% decrease but <25% increase)	65 [34.2%]	-
Progressive disease (PD, ≥25% increase)	8 [4.2%]	-
Absolute volume change in cm3 (mean, range)	−2.26	(−42.42/42.44)
Relative volume change (mean, range)	−29.6%	(−98.4%/190.5%)
Volume change per month (mean, range)	−0.85%	(−6.56%/7.62%)

Abbreviations: SRS, stereotactic radiosurgery; RT, radiotherapy; BED, biologically effective dose; SFED, single-fraction equivalent dose; Gy, gray.

### 2.3. Gamma Knife Treatment

Between 2011 and 2021, treatments were delivered with a Gamma Knife model 4C; thereafter, the model ICON (Elekta Instrument AB, Stockholm, Sweden) was used. The treatment technique has previously been described in detail [26]. On the day of treatment, MRI sequences were coregistered with a stereotactic 3D CT image set.

The prescribed margin dose, which depended on tumour size and location, ranged from 11 to 24 Gy (Table 1). The majority of meningiomas (165 cases) received a single session, with margin doses of 11 to 18 Gy (mean 13.7 Gy).

Per institutional protocol, tumours lying close to organs at risk (OARs), and, in particular, to the anterior optic pathway (AOP), were managed with hypofractionated stereotactic radiosurgery (HFSRS). Twenty-five cases were treated in this way, receiving either 6 Gy in three fractions or 5–6 Gy in four fractions on consecutive days.

To permit comparison across the different fractionation regimens, the biologically effective dose (BED) was derived from the widely used linear–quadratic (LQ) model, whose limitations at high doses are acknowledged [27]. HFSRS doses were additionally converted to single-fraction equivalent doses (SFEDs) [28] so that they could be compared with conventional single-session radiosurgery. When an α/β ratio of 3.76 Gy was applied [29], the margin SFED calculated for the HFSRS treatments fell between 9.4 and 13.5 Gy (mean 11.9 Gy).

Treatment planning was carried out on the Leksell GammaPlan workstation (Elekta AB), with the aim of achieving high tumour coverage (mean 96.3%) while restricting the dose to critical structures such as the anterior optic pathway, cochlea and brainstem.

### 2.4. Sample Size Calculation

Sample size was estimated prospectively from a pilot experiment in 100 patients. The calculation indicated that 136 patients, including 23 positive cases, would be needed to detect an AUC of 0.70 at α = 0.05 and β = 0.10 (MedCalc v20; MedCalc Software Ltd., Ostend, Belgium). These 100 pilot patients were retained in the final CE-T1w cohort of 190 patients, which included 45 positive cases.

### 2.5. MRI Acquisition and Tumour Delineation

Imaging was performed on a 3-Tesla scanner (Achieva; Philips, Eindhoven, The Netherlands). A 3D-T1w magnetisation-prepared rapid acquisition gradient echo (MPRAGE) sequence was acquired before and after intravenous contrast administration, with TR/TE/TI 6.8/3.2/900 ms, flip angle 8° and measured voxel size 0.6 × 0.6 × 1.0 mm.

### 2.6. Postprocessing

All 190 meningiomas were manually delineated on CE-T1w images on the Leksell GammaPlan workstation by neurosurgeons with 5 to 16 years of radiosurgical experience working with a neuroradiologist with more than 43 years of experience. Image sets were reviewed for quality, and sequences with artifacts were excluded. Segmentations were stored as three-dimensional masks corresponding to the pretreatment MRI volumes. Image and mask files were paired using exact canonical patient identifiers; ambiguous, duplicate or unusable patient identifiers were rejected rather than resolved by substring matching.

When image and mask geometry differed in matrix dimensions, voxel spacing, orientation or origin, the segmentation mask was resampled to the corresponding MRI grid using nearest-neighbour interpolation to preserve discrete mask labels. The MRI volume itself remained on its native acquisition grid at this stage.

### 2.7. Follow-Up

Imaging and clinical follow-up were performed every six months for the first two years after GKRS and yearly thereafter. Meningioma volumes were measured at every follow-up examination.

### 2.8. Study Design

The continuous endpoint was relative tumour volume change per base-10 logarithm of follow-up time. It accounts for the non-linear decline of tumour volume after SRS [12] and enables better comparison across long and heterogeneous follow-up periods compared to volume change per month:

Relative volume change per log_10_ (month) = (V_last − V_SRS)/(V_SRS × log_10_ (FU))

where V_SRS is the tumour volume at SRS, V_last is the volume at last follow-up, and FU is the time from SRS to last follow-up in months. The binary endpoint defined responders as a relative volume change of ≤−0.30 per log_10_ (month), corresponding to a cumulative decrease of approximately 32% at 12 months, 50% at the mean follow-up of 47.5 months and 65% at 152 months. Two thresholds were involved. The outcome threshold defined the actual responders. This cutoff was not derived from RANO criteria but rather chosen to identify approximately the strongest-responding quarter of the cohort, based on the hypothesis that these patients would show morphological clues typical of good responders. The second was the prediction threshold, which used development out-of-fold Youden cutoffs, applied unchanged to each holdout cohort. AUC, the primary performance measure, was calculated against the binary outcome and does not depend on the prediction threshold.

Patient identifiers were sorted in ascending numeric order, which reflects the order of treatment. Five chronological development/test splits were evaluated (168/22, 162/28, 156/34, 150/40 and 144/46 patients), with the six-patient increment chosen pragmatically to vary test-set size without deeply reducing the development cohort. These five splits share patients and cannot be considered to be entirely separate validation cohorts. In each split, model fitting, feature selection and hyperparameter optimisation used the development cohort only, after which the final model was refitted on the full development cohort and evaluated once on the holdout set. The workflow enforced patient-level separation and checked explicitly for duplicate identifiers and development/test overlap.

### 2.9. Analysis Code and Computational Environment

Starting from paired NRRD images and segmentation masks, all analyses were performed by a single executable Python notebook (Configurable-leakage-controlled-multi-workflow-medical-image-classification-suite, version 7.6) that implements the three workflows described in Section 2.13, Section 2.14 and Section 2.15, together with initial per-image preprocessing, cohort partitioning, training-fold-specific processing, feature selection, threshold determination and performance assessment, in a fixed order within a single run. For users starting from DICOM data, we also provide a separate script for the initial conversion of DICOM files into paired NRRD images and segmentation masks. The code is openly available (see Data Availability) and requires no local software installation or command-line use. It runs in Google Colab, where every user-configurable setting is collected and documented at the top of the file, with its location and effect listed in the header, so that the user needs only to update the path to the folder containing the images and their corresponding masks, select a runtime, and execute. The analyses were run on an NVIDIA T4 GPU high-RAM instance and completed in about four hours.

### 2.10. Image Intensity Normalisation and Tumour-Centred Preprocessing

Image intensities were standardised independently for each patient using the mean and standard deviation within the segmented tumour. The complete MRI volume was converted to z scores and clipped at ±2.5 standard deviations, and the clipped range was mapped linearly onto [0, 1], placing the tumour mean at 0.5. For each patient, the axial slice with the largest tumour cross-sectional area was selected for analysis.

A tumour-centred square crop including a two-pixel margin was generated using the in-plane voxel spacing, zero-padded when necessary and resized to 224 × 224 pixels by bilinear interpolation. The CE-T1w image was replicated across three identical channels. The segmentation mask was used for preprocessing and tumour localisation only and was not supplied to the network as an input channel.

### 2.11. Deep Learning Architectures and Pretraining

ResNet-10t (5 million parameters), DenseNet-121 (8 million), ResNet-18 (11.7 million) and ResNet-50 (25.6 million) with ImageNet or RadImageNet pretraining were evaluated in the end-to-end CNN workflows and as fixed feature extractors for downstream machine learning classification. ImageNet weights were obtained from the torchvision model repository, and RadImageNet weights were obtained from the published RadImageNet release [16]. For end-to-end training, each cross-validation fold was initialised independently from the same pretrained weights, with no transfer of trained weights, optimiser states or BatchNorm statistics between folds.

### 2.12. Patient-Level CNN Processing

The preprocessed single-slice input was passed through the CNN backbone and globally average-pooled to obtain one patient-level feature vector of 512 dimensions for ResNet-10t and ResNet-18, 1024 for DenseNet-121 and 2048 for ResNet-50. In the frozen end-to-end workflow, this vector was passed to a compact classification head comprising batch normalisation, dropout of 0.5 and one linear output unit. The fine-tuning workflow used a similarly compact head with layer normalisation and dropout of 0.30. The final output was one logit per patient, converted to a probability using the sigmoid function.

### 2.13. Workflow 1: Frozen End-to-End CNN Classification with Head-Only Training

Only the classification head was trained, with backbone weights and BatchNorm statistics kept fixed, under five-fold stratified patient-level cross-validation repeated twice. Training used AdamW optimisation (learning rate 1 × 10^−3^, weight decay 1 × 10^−4^), a batch size of 8, cosine learning-rate scheduling and class-weighted binary cross-entropy for at most 15 epochs, with early stopping after 10 epochs without improvement in validation AUC. The epoch with the highest validation AUC was retained in each fold. Training images were augmented by random horizontal and vertical flips, whereas validation and holdout images were not augmented. After cross-validation, a newly initialised head was refitted on the complete development cohort for the median number of epochs across folds.

### 2.14. Workflow 2: Frozen CNN Feature Extraction and ML Classification

The pretrained backbones served as fixed feature extractors, run in evaluation mode with gradient computation disabled and without augmentation. Gaussian naive Bayes and logistic regression were evaluated by nested stratified cross-validation within the development cohort (three outer and three inner folds), with every data-dependent step fitted on training folds only. Within each training fold, features underwent median imputation, near-zero-variance filtering and univariate AUC filtering, which retained features with sufficiently strong direct or inverse discrimination, the latter being directionally inverted. After z-score scaling and correlation filtering, the remaining features were evaluated by five selection methods: normalised and conventional mRMR, a Boruta-like random forest procedure with shuffled shadow features, and L1-regularised/elastic-net logistic regression. Features supported by at least three methods were retained, with a minimum feature set enforced when necessary, and class imbalance was then addressed by random oversampling. Logistic-regression hyperparameters were tuned in the inner loop, whereas naive Bayes was fitted without tuning, with AUC as the ranking metric. The selected workflow was refitted on the complete development cohort.

### 2.15. Workflow 3: Restricted CNN Fine-Tuning

Fine-tuning was evaluated using five-fold stratified patient-level cross-validation repeated three times. Within each outer training fold, a stratified 20% subset of the outer training patients was reserved for inner validation. This subset was used to select the backbone learning rate and the retained numbers of head training and fine-tuning epochs. Outer-validation patients were used only for performance estimation. Epoch selection was governed by inner-validation AUC smoothed using a three-epoch trailing mean. The checkpoint with the highest raw AUC within the selected smoothing window was retained, with log loss used to resolve AUC ties.

Training used AdamW optimisation, a batch size of 8 and class-weighted binary cross-entropy, with the positive-class weight calculated from the corresponding training data. In the first phase, the classification head was trained on the frozen backbone for up to 20 epochs using a learning rate of 1 × 10^−3^ and weight decay of 1 × 10^−4^. During inner selection, this phase used early stopping after six epochs without improvement and cosine learning-rate decay to 5% of the initial rate.

In the second phase, an architecture-specific terminal portion comprising approximately 19–25% of the backbone parameters (ResNet-10t, conv1 of the final layer4 block; ResNet-18, conv2 of the final layer4 block; ResNet-50, the final layer4 bottleneck; DenseNet-121, transition3 and the last six denseblock4 layers) was unfrozen in a single stage and trained jointly with the head for up to 15 epochs. The head learning rate was 1 × 10^−4^, whereas the backbone learning rate was selected within each fold from architecture-specific candidates ranging from 5 × 10^−6^ to 5 × 10^−5^. Weight decay was 1 × 10^−4^ for the head and 1 × 10^−5^ for the backbone. The second phase used a three-epoch warm-up followed by cosine decay to 5% of the peak learning rate and, during inner selection, early stopping after five epochs without improvement. Stochastic depth with a drop-path rate of 0.05 was applied only to ResNet-10t.

Movement of the backbone away from its pretrained state was constrained using an L2-SP penalty (λ = 1 × 10^−4^). Fine-tuning, including the final refit, was halted when cumulative movement exceeded 3% relative RMS, although the epoch crossing this threshold remained eligible for selection. BatchNorm running statistics and affine parameters remained fixed, except for the final norm5 affine parameters of DenseNet-121. Training images were augmented using random horizontal and vertical flips and rotations of up to ±12 degrees. Validation and holdout images were not augmented, and no test-time augmentation was used.

After cross-validation, a single fresh model was initialised from the pretrained weights and refitted on the complete development cohort. The most frequently selected protocol across the outer-fold runs was used, together with the median selected numbers of head training and fine-tuning epochs among the folds supporting that protocol.

### 2.16. Performance Assessment and Sensitivity to Contour Variation

Holdout AUC uncertainty was assessed using DeLong and delete-one-patient jackknife normal-approximation 95% confidence intervals and a stratified patient-level nonparametric bootstrap percentile 95% confidence interval, with 2000 resamples drawn separately within each outcome class. The jackknife analysis also recorded the minimum and maximum leave-one-out AUC values to quantify dependence on individual holdout patients.

Threshold-dependent measures included accuracy, balanced accuracy, sensitivity, specificity, positive predictive value (precision), F1 score and Matthews correlation coefficient. The Brier score was also calculated as a threshold-independent measure of probabilistic performance. Threshold-dependent measures were calculated at a cutoff derived exclusively from development out-of-fold predictions and applied unchanged to the holdout cohort: Youden’s J on pooled out-of-fold predictions for Workflows 1 and 2, and the median of the three repeat-wise out-of-fold Youden thresholds for Workflow 3.

Sensitivity to contour variation was assessed using the fine-tuned ResNet-10t and the 156/34 development/holdout split. First, SAM 2 generated new segmentations automatically, guided by rectangular boxes derived from the manual contours on every tumour-containing axial slice. The model was then retrained using the development cohort before holdout evaluation. Second, holdout test masks were subjected to dilation and erosion by 1–3 voxels, translations of 2 or 5 mm, and isotropic linear scaling by ±10% or ±20% about the mask centre of mass. Scaling used nearest-neighbour interpolation on the original grid. Percentages refer to linear dimensions rather than tumour volume. Preprocessing was repeated using the modified masks and the reference model was evaluated without retraining. For translations, AUCs were averaged across five random directions, whereas ΔAUC was calculated from direction-averaged patient probabilities. Contour agreement was quantified by the Dice similarity coefficient against manual contours using the combined development and holdout cohorts for SAM 2 and the holdout cohort alone for simulated perturbations.

## 3. Results

Tumour control was achieved in 95.8% of meningiomas. Mean follow-up was 47.5 months (range, 12–152). According to RANO criteria [30], 40 patients (21.1%) showed a partial response, 77 (40.5%) showed a minor response, 65 (34.2%) showed stable disease and 8 (4.2%) showed progressive disease. Mean tumour volume decreased by 2.26 cm^3^, corresponding to a mean reduction of 0.85% per month (Table 1).

Three complementary modelling strategies were evaluated (Figure 1)—frozen end-to-end CNN classification (Workflow 1), frozen CNN feature extraction followed by machine learning classification (Workflow 2), and restricted CNN fine-tuning (Workflow 3)—all trained and optimised within the development cohort and evaluated once on the untouched holdout set.

Within the five development/test splits, the highest median discrimination was observed for fine-tuned RadImageNet ResNet-10t (median AUC 0.836, range 0.738 to 1.000), which also produced the two highest individual AUCs (1.000 and 0.953) across all workflows and splits (Table 2, Figure 2a,b).

Although the smallest CNN achieved the highest median AUC, backbone size did not consistently predict performance (Table 2). Under the frozen strategy, the smallest RadImageNet ResNet-10t gave the lowest median AUC (0.735), below that of both the larger ResNet-50 (0.808) and DenseNet-121 (0.804) (Table 2). Architecture and training strategy also interacted: fine-tuning outperformed freezing for RadImageNet ResNet-10t, whereas freezing was superior for DenseNet-121 and ResNet-50. While RadImageNet-pretrained models were frequently among the top performers, the benefit of radiology-specific pretraining varied by architecture (Table 2). Fine-tuned ResNet-10t reached the highest peaks but exhibited substantial variability (AUC 0.738–1.000; MCC 0.215–1.000), whereas the feature-based ResNet-10t + NB (0.760–0.846) and LR (0.800–0.871) workflows maintained comparatively narrow AUC ranges, with lower peak and median AUCs (Figure 2a). Across the splits, the AUC trajectory of fine-tuned ResNet-10t closely followed that of frozen DenseNet-121 (Pearson r = 0.966), which also tracked its NB (r = 0.950) and LR (r = 0.944) counterparts. Because the five splits overlap, such correlations should be interpreted with caution. This trade-off between peak discrimination and split-to-split robustness was consistent across the highest-ranked models, which supports the consideration of both median AUC and its range when evaluating model performance (Figure 2a).

Predictive performance was highly sensitive to the size of the development and test sets. Fine-tuned RadImageNet ResNet-10t achieved the overall highest AUCs in the splits with the largest development sets (n = 168, 162 and 156) (Table 2 and Table 3; Figure 2a,b). Conversely, in the splits with the largest test sets (n = 46 and 40), feature-based classifiers achieved the highest AUCs (Table 2; Figure 2a). Specifically, ResNet-10t features classified by NB stood out in the 144/46 and 150/40 splits, achieving AUCs of 0.820 and 0.846, with MCCs of 0.507 and 0.649, respectively (Table 2 and Table 3).

As shown in Table 3, correlations between AUC and threshold-dependent measures ranged from −0.14 to 0.93. Associations were generally strongest for ResNet-10t + NB, particularly for balanced accuracy (r = 0.93), MCC (r = 0.90) and NPV (r = 0.89). Fine-tuned ResNet-10t showed weak associations with accuracy (r = 0.06) and specificity (r = −0.14), which indicates that stronger ranking performance did not consistently translate into better classification at the development-derived threshold. Frozen ResNet-50 showed positive correlations ranging from 0.31 to 0.62. These correlations are descriptive because they are based on only five overlapping splits.

We assessed sensitivity to tumour contour variation in the 156/34 development/holdout split, chosen because it lies in the middle of the five chronological splits, using two approaches (Table 4). First, manual contours were replaced by SAM 2 segmentations generated automatically with guidance from the manual contours, and the model was retrained to evaluate the pipeline with an alternative segmentation method. Second, manual contours were altered by predefined amounts, and the reference model was re-scored without retraining to assess sensitivity to simulated contour errors. Replacing manual contours with SAM 2 segmentations and retraining yielded a holdout AUC of 0.816, compared with 0.836 for manual contours (paired DeLong *p* = 0.90), at a median DSC of 0.91. Simulated contour errors reduced AUC, with scaling producing the largest reduction and erosion producing the smallest. AUC remained at or above 0.650 across all tested conditions, with a maximum reduction of 0.186 under scaling. The lowest median DSC, 0.58, occurred under shifting and was below the reported inter-observer range of 0.85–0.93 for meningioma delineation. For simulated contour errors, the DSC, AUC and ΔAUC ranges in Table 4 represent variation across perturbation levels rather than confidence intervals; their endpoints do not necessarily correspond to the same perturbation level.

## 4. Discussion

The main findings suggest that pretreatment MRI can discriminate meningioma response to GKRS in a relatively small cohort, but performance depends strongly on CNN architecture, pretrained weights, training strategy and development/test partition, with a trade-off between peak discrimination and consistency across splits.

The best median AUC in the present study (0.836) was comparable with the 0.81–0.84 reported by previous meningioma radiomics models [11,12], while our individual splits reached 1.000 and 0.953. These peak values should be considered alongside the AUCs across all five splits, with an emphasis on the median performance. Such very high AUCs in individual splits should not be interpreted as evidence of superiority without validation in a larger external cohort. Li et al. reported the first deep learning study of post-GKS outcome prediction in meningioma, evaluating 38 models for peritumoural oedema in 595 patients [31]. C-indices ranged from 0.564 to 0.886 internally (median 0.742) and from 0.520 to 0.780 externally (median 0.685). The endpoint in the Li et al. study was time to post-GKS peritumoural oedema development, not meningioma volume reduction. The study did not describe nested validation or explicitly address information leakage.

The highest AUCs in the present study occurred in smaller test sets and should therefore be interpreted cautiously. Small test sets yield less precise performance estimates and increase the likelihood of unusually high or low AUCs through sampling variation [8,32]. A smaller test set necessarily meant a larger development set, so the stronger performance may partly reflect the additional training data. This pattern was apparent for ResNet-10t, whose strongest fine-tuned performance occurred in the three splits with the largest development cohorts, whereas fixed ResNet-10t features classified by NB performed well even when fewer patients were available for development. This is consistent with previous findings that fixed pretrained features combined with conventional machine learning classifiers can outperform fine-tuning in small imaging cohorts [7,33,34,35]. While the above suggests that deep learning benefits from more training data, the results are inconclusive due to simultaneous changes in test-set size and composition.

An additional finding was that the effect of fine-tuning on median AUC was architecture-dependent, improving RadImageNet ResNet-10t, whereas DenseNet-121 and ResNet-50 achieved higher median AUCs when frozen. Similar architecture-dependent responses have been reported previously, with different fine-tuning strategies varying in effectiveness across ResNet, DenseNet and VGG architectures and no single strategy proving consistently optimal [36,37]. In the present study, fine-tuning was deliberately restricted to a small upper portion of each backbone to limit the number of trainable parameters. Because fine-tuning depth was not systematically varied, these results demonstrate architecture-dependent performance under this restricted strategy rather than identifying the optimal extent of fine-tuning for each CNN. Previous work indicates that optimal fine-tuning depends on the size of the medical imaging cohort, its similarity to the pretraining data, and the CNN architecture [7,38,39,40].

Radiology-specific pretraining improved predictive performance but not reliably: ImageNet was better for fine-tuned ResNet-18 and for frozen ResNet-10t, while RadImageNet was better for DenseNet-121 and ResNet-50. Its predictive value therefore depended on both architecture and training strategy. This is consistent with evidence that transfer learning performance in small datasets depends strongly on the pretraining source. Models pretrained on images from the same anatomical site mostly outperform those pretrained on less related data [41], and disease-related pretraining has also outperformed natural-image pretraining [42].

The five temporal splits should be viewed as a robustness analysis across chronological development/test cutoffs, which places the emphasis on the median AUC across splits rather than on any single result. They are not independent validation cohorts or a hunt for a lucky outlier. Examining several development/test partitions can reveal the extent to which performance depends on the position of the chronological cut, an important consideration in small datasets [8]. Pan et al. assessed stability by repeating stratified random five-fold cross-validation ten times, whereas Liu et al. evaluated performance across the ten test folds of randomly shuffled, subject-level stratified ten-fold cross-validation [43,44]. Chronological evaluation also reflects clinical use more closely because models developed on earlier patients are applied to later patients and may reveal temporal changes in imaging, patient characteristics or clinical practice [45]. In brain metastases, the pretreatment-only AUC of 0.672 reported by Cho et al. declined to 0.595 in a chronologically later institutional cohort [13], which illustrates how strongly chronological evaluation can reduce performance.

Several methodological choices were intended to limit overfitting. The first was to analyse a single tumour-centred 2D axial slice per patient. A 2D backbone requires fewer trainable parameters than a volumetric model and permits the direct use of established 2D ImageNet- and RadImageNet-pretrained architectures. Although 3D networks preserve more complete spatial context and may outperform 2D approaches in some MRI tasks [46], no universally superior 2D or 3D strategy has been established [47]. Selecting the largest-area slice maximised tumour content. Keeping tumour predominance in peripheral slices would require progressively greater zoom as the tumour cross-section diminishes, which would introduce scale inconsistency across the dataset. An alternative is to sample axial, coronal and sagittal planes through the tumour centre, which would provide near-maximal cross-sections in all three planes [48]. Published multi-slice approaches commonly apply a crop defined on the largest cross-section to peripheral slices as well, while the resulting tumour proportion is rarely quantified [15,48,49].

The contour-variation analysis showed that segmentation choices can influence prediction even when the mask is not supplied directly to CNN, because contours determine intensity normalisation, slice selection and cropping. Retraining with SAM 2 contours produced a similar holdout AUC. SAM 2 segmentation was not fully independent, as manual contours were used as a guide. With the reference model held fixed, scaling produced the largest AUC reduction and erosion produced the smallest, which suggests that sensitivity depends on the type of contour alteration rather than overlap alone. The scaling perturbations were substantial: a 20% change in linear dimensions corresponds theoretically to an approximately 49% volume reduction or to 73% enlargement. These findings characterise sensitivity under the tested conditions, but evaluation of one model and one development/holdout split cannot establish robustness to the full range of contour variation encountered in clinical practice.

Augmentation was deliberately conservative, limited to flips and small rotations and restricted to training data. Stronger transformations could alter tumour morphology, margins, texture or signal characteristics carrying prognostic information. Flip-only augmentation has been used in brain MRI to avoid shape distortion, while excessive augmentation was reported to introduce bias or overfitting [7]. Piffer et al. noted that conventional augmentation produces highly correlated variants rather than independent observations and may alter relevant image features [1].

Strict leakage control was implemented at two levels. First, patient-level separation was guaranteed by design, because each patient contributed a single image and thus could not be assigned to both development and test sets unless duplicated. Assigning multiple images from the same tumour across training and test sets can produce optimistically biased performance estimates [15]. Second, feature selection posed a separate leakage risk. Frozen CNNs yield far more features than there are patients, so some features separate the classes by chance, and selecting them on data later used for testing carries this chance separation also into the performance estimate, making it overoptimistic. All feature ranking and consensus selection were therefore confined to training folds within a fully nested design [50,51]. Beyond leakage control, consensus feature selection was conducted to reduce dependence on any single selection method.

The openly shared code addresses the limited reproducibility of small-data medical imaging studies. In a systematic review of 77 studies, complete code for preprocessing, modelling and evaluation was available in only 13 (17%), and incomplete methodological reporting was identified as an important barrier to the assessment of potential bias [1]. The present study supplies that complete chain as a single executable script.

Several limitations remain. The cohort was relatively small and derived from a single institution. Because of the small cohort size, the holdout sets partially overlapped and, thus, the resulting AUC ranges are not independent. However, because all workflows were evaluated on identical test sets, this design still allowed direct comparison between workflows and provided an estimate of robustness, which studies of small cohorts rarely attempt. Chronological testing cannot substitute for external validation, which often reveals performance below that seen in internal testing [1,52,53]. The findings should therefore be considered proof of concept pending multicentre validation. The analysis was restricted to contrast-enhanced T1-weighted MRI, although combined structural and functional MRI features have outperformed single-modality approaches in a recent small-sample study [44]. Including additional sequences would, however, have reduced our effective cohort size because they were not available for every patient. Threshold-dependent measures used development out-of-fold Youden cutoffs applied unchanged to holdout, permitting individual-patient classification. In small cohorts, uncertainty in these thresholds and imprecision in holdout estimates can contribute to discrepancies between discrimination and classification performance: fine-tuned ResNet-10t achieved an AUC of 0.953 in the 168/22 split but an accuracy of 0.364 and MCC of 0.215. This highlights the importance of assessing threshold-dependent performance alongside AUC. Finally, most tumours were presumed WHO grade I meningiomas based on imaging alone, so higher-grade meningiomas or alternative diagnoses cannot be entirely excluded.

## 5. Conclusions

To our knowledge, this is the first study to apply deep learning to predict volumetric meningioma response following Gamma Knife radiosurgery. No single strategy achieved both the highest AUCs and the greatest consistency across chronological partitions. Chronological partitioning assessed performance in patients treated later than those used for model development, approximating prospective use. In small cohorts, consistency across partitions may matter as much as the highest AUC from one test split. The principal contribution of this work is the openly provided leakage-controlled pipeline, which brings together three transfer learning strategies: fine-tuning, training with frozen backbone weights, and feature extraction followed by machine learning classification. The pipeline also includes chronological split control and a direct comparison of RadImageNet and ImageNet pretraining. It enables these configurations to be evaluated under identical conditions in a single Google Colab notebook, with user-configurable parameters collected in one block so that researchers without programming experience can apply it to their own cohorts.

## Figures and Tables

**Figure 1:**
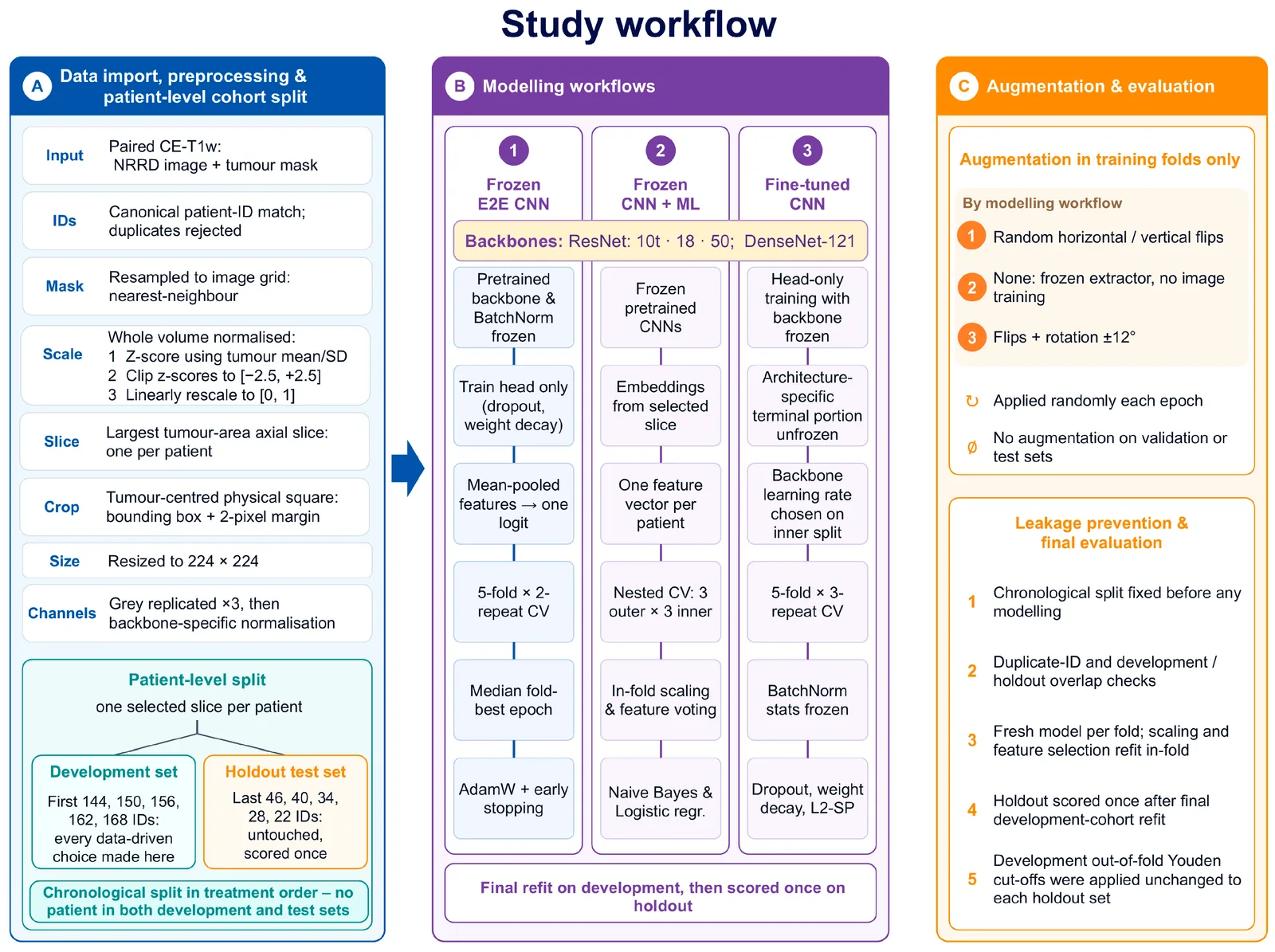
Workflows of deep learning and machine learning ranking and classification analyses. Pre-GKRS MRI scans were acquired, and tumour VOIs were segmented. Before feature extraction, images were z-score-normalised, interpolated and resampled; one tumour-containing axial slice per patient was then selected and assigned to fixed development or holdout test sets. Three modelling strategies were evaluated: frozen CNN feature extraction followed by machine learning, frozen end-to-end CNN classification, and CNN fine-tuning. Model development and optimisation were performed exclusively within the development cohort, with augmentation restricted to training folds. Final models were refit on the full development cohort and evaluated once on the untouched holdout test set. Threshold-dependent performance was calculated using the development out-of-fold Youden cutoffs, applied unchanged to each holdout cohort. Abbreviations: E2E, end-to-end.

**Figure 2:**
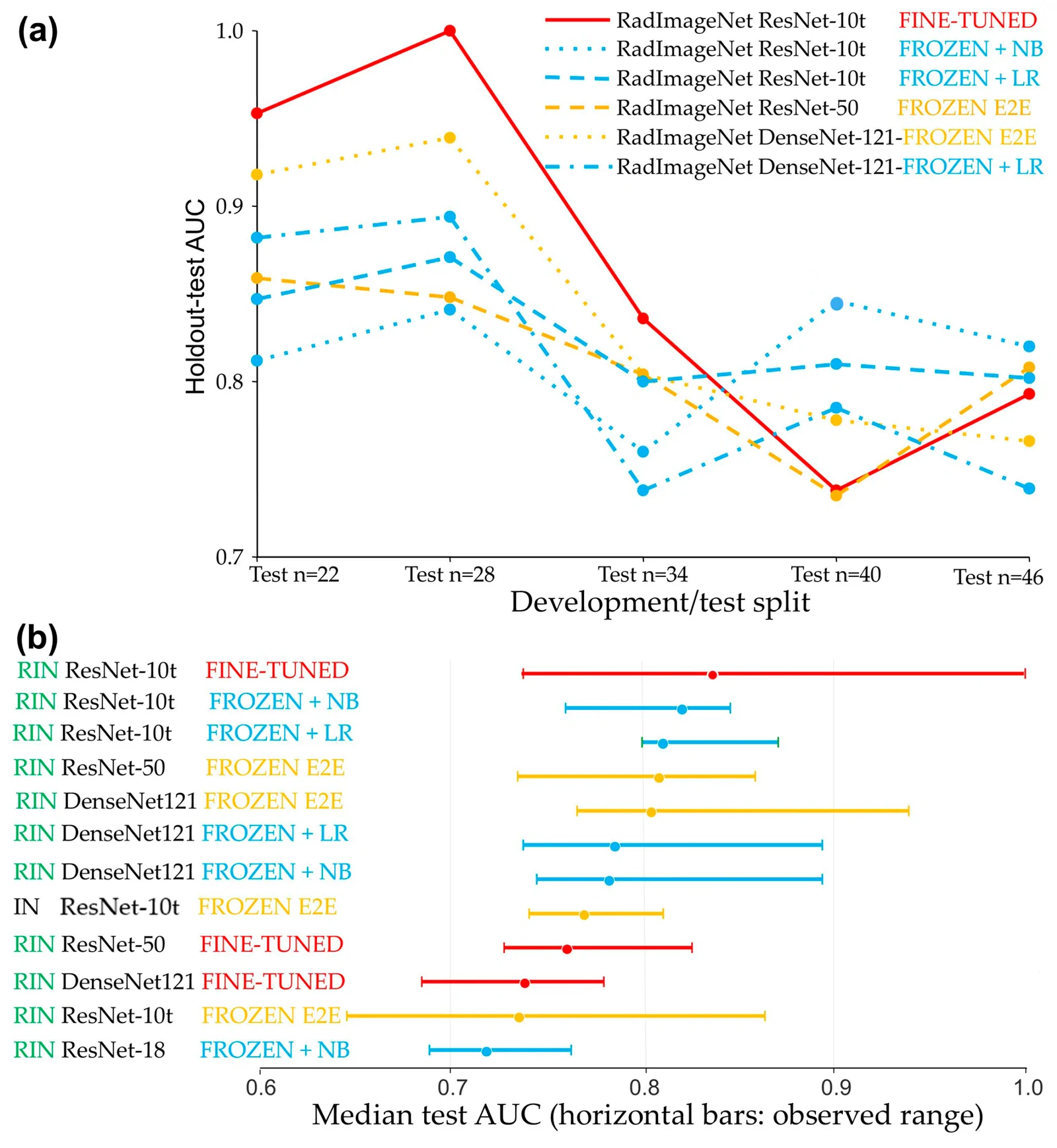
Performance trajectories and stability of classification models across chronological development/test partitions. (**a**) AUC trajectories (in test sets) of the six highest-ranked models across five development/test splits, illustrating marked differences in performance. (**b**) Summary distribution of the median holdout test AUCs for the 12 highest-ranked models across the same development/test splits, with horizontal bars indicating the absolute observed performance range. The panels show a predominance of RadImageNet-pretrained models among the best performers and a trade-off between peak discrimination performance and split-to-split robustness. While fine-tuned models achieved the highest individual and median AUCs, feature-based workflows utilising NB or LR maintained narrower performance ranges across splits. Because the five splits share patients, the ranges shown reflect sensitivity to the chronological cutoff rather than independent validation. Abbreviations: RIN: RadImageNet; IN: ImageNet.

**Table 2:** Model performance across development–test splits ^a,b^.

Model/Strategy ^c^	Dev. 168/Test 22 5 Positives	Dev. 162/Test 28 6 Positives	Dev. 156/Test 34 9 Positives	Dev. 150/Test 40 9 Positives	Dev. 144/Test 46 9 Positives	Across Splits
	AUC 95% CI MCC	AUC 95% CI MCC	AUC 95% CI MCC	AUC 95% CI MCC	AUC 95% CI MCC	Median AUC range
CNN: RadImageNet ResNet-10t fine-tuned	0.953 0.868–1.000 0.215	1.000 1.000–1.000 1.000	0.836 0.635–1.000 0.597	0.738 0.522–0.955 0.339	0.793 0.592–0.993 0.264	0.836 0.738–1.000
CNN + ML: RadImageNet ResNet-10t + NB	0.812 0.561–1.000 0.482	0.841 0.643–1.000 0.471	0.760 0.518–1.000 0.265	0.846 0.669–1.000 0.649	0.820 0.624–1.000 0.507	0.820 0.760–0.846
CNN + ML: RadImageNet ResNet-10t + LR	0.847 0.634–1.000 0.561	0.871 0.710–1.000 0.427	0.800 0.588–1.000 0.333	0.810 0.589–1.000 0.355	0.802 0.584–1.000 0.507	0.810 0.800–0.871
CNN: RadImageNet DenseNet-121 frozen	0.918 0.749–1.000 0.495	0.939 0.816–1.000 0.522	0.804 0.602–1.000 0.335	0.778 0.568–0.988 −0.020	0.766 0.564–0.968 0.456	0.804 0.766–0.939
CNN: RadImageNet ResNet-50 frozen	0.859 0.679–1.000 0.561	0.848 0.652–1.000 0.427	0.804 0.645–0.964 0.333	0.735 0.544–0.925 0.385	0.808 0.678–0.938 0.428	0.808 0.735–0.859
CNN + ML: RadImageNet DenseNet-121 + LR	0.882 0.671–1.000 0.583	0.894 0.683–1.000 0.386	0.738 0.484–0.991 0.164	0.785 0.557–1.000 0.221	0.739 0.495–0.982 0.186	0.785 0.738–0.894
CNN + ML: RadImageNet DenseNet-121 + NB	0.835 0.608–1.000 0.325	0.894 0.713–1.000 0.471	0.782 0.554–1.000 0.351	0.778 0.592–0.964 0.327	0.745 0.504–0.985 0.151	0.782 0.745–0.894
CNN: ImageNet ResNet-10t frozen	0.741 0.425–1.000 0.376	0.811 0.572–1.000 0.427	0.809 0.644–0.973 0.369	0.753 0.548–0.957 0.342	0.769 0.590–0.947 0.341	0.769 0.741–0.811
CNN: RadImageNet ResNet-50 fine-tuned	0.788 0.558–1.000 0.277	0.826 0.625–1.000 0.440	0.760 0.551–0.969 0.272	0.728 0.532–0.923 0.247	0.748 0.575–0.920 0.366	0.760 0.728–0.826
CNN: RadImageNet ResNet-10t frozen	0.765 0.482–1.000 0.588	0.864 0.700–1.000 0.503	0.662 0.434–0.890 0.354	0.735 0.506–0.964 0.431	0.646 0.415–0.876 0.207	0.735 0.646–0.864
CNN: RadImageNet DenseNet-121 fine-tuned	0.718 0.367–1.000 0.399	0.780 0.500–1.000 0.440	0.738 0.496–0.980 0.491	0.685 0.443–0.926 0.431	0.754 0.552–0.956 0.232	0.738 0.685–0.780
CNN + ML: RadImageNet ResNet-18 + NB	0.718 0.442–0.994 0.215	0.727 0.438–1.000 0.438	0.689 0.466–0.912 0.164	0.763 0.594–0.933 0.137	0.700 0.494–0.905 0.232	0.718 0.689–0.763
CNN: ImageNet ResNet-50 fine-tuned	0.706 0.396–1.000 0.482	0.765 0.472–1.000 0.503	0.698 0.445–0.950 0.396	0.670 0.419–0.921 0.358	0.700 0.465–0.934 0.254	0.700 0.670–0.765

^a^ Holdout test results from the final refit. AUC 95% CIs: DeLong, normal approximation, truncated at 1.000. MCC at the development out-of-fold Youden threshold (Section 2.16). ^b^ Only models with median AUC across the five splits ≥0.70 are shown. ^c^ All models are presented in Table S1. CNN frozen, only the classification head trained; CNN fine-tuned, head trained first, then the final backbone segment (~20–25% of weights; BatchNorm frozen); CNN + ML, frozen CNN features classified by LR or NB. Abbreviations: AUC, area under the receiver-operating-characteristic curve; CI, confidence interval; MCC, Matthews correlation coefficient; NB, naive Bayes; LR, logistic regression.

**Table 3:** Detailed metrics for the best-performing model (by median AUC) from each of three workflows across five development/test splits ^a^.

Split ^b^	AUC	95% CI ^c^	Threshold	Acc.	Bal. Acc.	Sensitivity	Specificity	PPV	NPV	MCC
**Fine-tuned CNN: RadImageNet ResNet-10t (median AUC: 0.84; range: 0.74–1.00)**										
168/22	0.95	0.87–1.00	0.47	0.36	0.59	1.00	0.18	0.26	1.00	0.22
162/28	1.00	1.00–1.00	0.47	1.00	1.00	1.00	1.00	1.00	1.00	1.00
156/34	0.84	0.64–1.00	0.52	0.85	0.76	0.56	0.96	0.83	0.86	0.60
150/40	0.74	0.52–0.96	0.52	0.80	0.63	0.33	0.94	0.60	0.83	0.34
144/46	0.79	0.59–0.99	0.47	0.52	0.66	0.89	0.43	0.28	0.94	0.26
**rAUC** ^d^	-		-	0.06	0.56	0.80	−0.14	0.28	0.85	0.54
**Frozen CNN feature extraction + ML: RadImageNet ResNet-10t + NB (median AUC: 0.82; range: 0.76–0.85)**										
168/22	0.81	0.56–1.00	0.40	0.82	0.74	0.60	0.88	0.60	0.88	0.48
162/28	0.84	0.64–1.00	0.09	0.75	0.78	0.83	0.73	0.45	0.94	0.47
156/34	0.76	0.52–1.00	0.01	0.59	0.65	0.78	0.52	0.37	0.87	0.26
150/40	0.85	0.67–1.00	0.19	0.85	0.86	0.89	0.84	0.62	0.96	0.65
144/46	0.82	0.62–1.00	0.54	0.80	0.79	0.78	0.81	0.50	0.94	0.51
**rAUC** ^d^	-		-	0.84	0.93	0.37	0.75	0.66	0.89	0.90
**Frozen backbone CNN: RadImageNet ResNet-50 (median AUC: 0.81; range: 0.74–0.86)**										
168/22	0.86	0.6 > 0.8–1.00	0.46	0.82	0.81	0.80	0.82	0.57	0.93	0.56
162/28	0.85	0.65–1.00	0.47	0.71	0.76	0.83	0.68	0.42	0.94	0.43
156/34	0.80	0.65–0.96	0.41	0.65	0.69	0.78	0.60	0.41	0.88	0.33
150/40	0.74	0.54–0.93	0.48	0.70	0.73	0.78	0.68	0.41	0.91	0.38
144/46	0.81	0.68–0.94	0.47	0.70	0.77	0.89	0.65	0.38	0.96	0.43
**rAUC** ^d^	-		-	0.53	0.59	0.31	0.48	0.54	0.34	0.62

^a^ Each workflow shows the model with the highest median AUC across five splits. Development OOF Youden thresholds were applied unchanged to holdout: median repeat-wise outer OOF thresholds for the fine-tuned model and pooled OOF thresholds for the others. ^b^ Split denotes development/test sample sizes. Splits partially overlap. ^c^ 95% CIs refer to AUC and use single-model DeLong variance. ^d^ Correlation rows: Pearson r of AUC against each threshold-dependent metric across the 5 splits, descriptive only. Abbreviations: AUC, area under the receiver-operating-characteristic curve; CI, confidence interval; Acc., accuracy; Bal. acc., balanced accuracy; MCC, Matthews correlation coefficient; CNN, convolutional neural network; NB, naive Bayes; NPV, negative predictive value; PPV, positive predictive value; ML, machine learning.

**Table 4:** Sensitivity of holdout AUC to tumour contour variation using SAM 2 re-segmentation guided by manual contours and simulated contour errors (fine-tuned ResNet-10t; development n = 156, holdout n = 34).

Contour	Median DSC vs. Manual	Holdout AUC	ΔAUC vs. Manual ^a^
Manual contour (reference)	1.00	0.836 (0.64 to 1.00)	Reference
Automatic segmentation guided by manual contours (model retrained)			
SAM 2, box prompt on every slice	0.91	0.816 (0.62 to 1.00)	−0.020
Simulated contour error (reference model re-scored)			
Dilation, 1 to 3 voxels	0.78 to 0.92	0.739 to 0.782	−0.097 to −0.054
Erosion, 1 to 3 voxels	0.72 to 0.91	0.795 to 0.816	−0.041 to −0.020
Shift, 2 to 5 mm	0.58 to 0.81	0.709 to 0.719 ^b^	−0.127 to −0.117
Scaling, ±10 to 20%	0.68 to 0.86	0.650 to 0.769	−0.186 to −0.067

^a^ ΔAUC relative to the manual contour reference. ^b^ Shift AUCs are averaged across five directions; ΔAUC uses direction-averaged probabilities. AUC, area under the ROC curve; parentheses indicate 95% DeLong confidence intervals. DSC, Dice similarity coefficient versus manual contours: SAM 2 uses the whole cohort (development and holdout sets, n = 190); simulated perturbations use the holdout set only (n = 34). Simulated-error entries show ranges across perturbation levels, not confidence intervals; endpoints need not correspond.

## Data Availability

The data and code used in this study are available in a publicly accessible GitHub repository at the following URL (when copying it into a browser, omit the sentence-ending period): https://github.com/markoradulovic-research/configurable-leakage-controlled-multi-workflow-medical-image-classification-suite/tree/main (accessed on 4 November 2026).

## Supplementary Materials

The following supporting information can be downloaded at: https://www.mdpi.com/article/10.3390/cancers18213590/s1, Supplementary Materials: Table S1. Model performance across all development-test splits ^a,b^.

## Abbreviations

3D	three-dimensional
AOP	anterior optic pathway
AUC	area under the curve
BED	biologically effective dose
CE-T1w	contrast-enhanced T1-weighted
CT	computed tomography
GKRS	Gamma Knife radiosurgery
HFSRS	hypofractionated stereotactic radiosurgery
LR	logistic regression
LQ,	linear–quadratic
MCC	Matthews correlation coefficient
ML	machine learning
MRI	magnetic resonance imaging
MPRAGE	magnetisation-prepared rapid acquisition gradient echo
OAR	organ at risk
RANO	Response Assessment in Neuro-Oncology
SFED	single-fraction equivalent dose
SGD	stochastic gradient descent
SRS	stereotactic radiosurgery
STROBE	Strengthening the Reporting of Observational Studies in Epidemiology
TR	repetition time
TE	echo time
TI	inversion time
VOI	volume of interest
WHO	World Health Organization
