# Supplementary material for: Realistic Performance Estimation in Small-Cohort Deep Learning and Machine Learning: A Leakage-Controlled Codebase Applied to Meningioma Response Prediction After Gamma Knife Radiosurgery
Source: Cancers (Basel). 2026 Nov 9;18(21):3590. doi: 10.3390/cancers18213590 (PMC13761412; doi:10.3390/cancers18213590)
Supplement: Supplementary file 1 [file cancers-18-03590-s001.zip › cancers-4619765-supplementary.pdf]

Table S1. Model performance across all development-test splits [a, b]

Model / strategy	Dev. 168 / Test 22<br>5 positives	Dev.162 / Test 28<br>6 positives	Dev. 156 / Test 34<br>9 positives	Dev.150 / Test 40<br>9 positives	Dev. 144 / Test 46<br>9 positives	Across<br>splits
	AUC<br>95% CI<br>MCC	AUC<br>95% CI<br>MCC	AUC<br>95% CI<br>MCC	AUC<br>95% CI<br>MCC	AUC<br>95% CI<br>MCC	Median AUC<br>range
CNN: RadImageNet ResNet-10t fine-tuned	0.953<br>0.868-1.000<br>0.215	1.000<br>1.000-1.000<br>1.000	0.836<br>0.635-1.000<br>0.597	0.738<br>0.522-0.955<br>0.339	0.793<br>0.592-0.993<br>0.264	0.836<br>0.738-1.000
CNN: RadImageNet DenseNet-121 frozen	0.918<br>0.749-1.000<br>0.495	0.939<br>0.816-1.000<br>0.522	0.804<br>0.602-1.000<br>0.335	0.778<br>0.568-0.988<br>-0.020	0.766<br>0.564-0.968<br>0.456	0.804<br>0.766-0.939
CNN + ML: RadImageNet DenseNet-121 + LR	0.882<br>0.671-1.000<br>0.583	0.894<br>0.683-1.000<br>0.386	0.738<br>0.484-0.991<br>0.164	0.785<br>0.557-1.000<br>0.221	0.739<br>0.495-0.982<br>0.186	0.785<br>0.738-0.894
CNN + ML: RadImageNet ResNet-10t + LR	0.847<br>0.634-1.000<br>0.561	0.871<br>0.710-1.000<br>0.427	0.800<br>0.588-1.000<br>0.333	0.810<br>0.589-1.000<br>0.355	0.802<br>0.584-1.000<br>0.507	0.810<br>0.800-0.871
CNN + ML: RadImageNet ResNet-10t + NB	0.812<br>0.561-1.000<br>0.482	0.841<br>0.643-1.000<br>0.471	0.760<br>0.518-1.000<br>0.265	0.846<br>0.669-1.000<br>0.649	0.820<br>0.624-1.000<br>0.507	0.820<br>0.760-0.846
CNN: RadImageNet ResNet-50 frozen	0.859<br>0.679-1.000<br>0.561	0.848<br>0.652-1.000<br>0.427	0.804<br>0.645-0.964<br>0.333	0.735<br>0.544-0.925<br>0.385	0.808<br>0.678-0.938<br>0.428	0.808<br>0.735-0.859
CNN + ML: RadImageNet DenseNet-121 + NB	0.835<br>0.608-1.000<br>0.325	0.894<br>0.713-1.000<br>0.471	0.782<br>0.554-1.000<br>0.351	0.778<br>0.592-0.964<br>0.327	0.745<br>0.504-0.985<br>0.151	0.782<br>0.745-0.894
CNN: ImageNet ResNet-10t frozen	0.741<br>0.425-1.000<br>0.376	0.811<br>0.572-1.000<br>0.427	0.809<br>0.644-0.973<br>0.369	0.753<br>0.548-0.957<br>0.342	0.769<br>0.590-0.947<br>0.341	0.769<br>0.741-0.811
CNN: RadImageNet ResNet-50 fine-tuned	0.788<br>0.558-1.000<br>0.277	0.826<br>0.625-1.000<br>0.440	0.760<br>0.551-0.969<br>0.272	0.728<br>0.532-0.923<br>0.247	0.748<br>0.575-0.920<br>0.366	0.760<br>0.728-0.826
CNN: RadImageNet ResNet-10t frozen	0.765<br>0.482-1.000<br>0.588	0.864<br>0.700-1.000<br>0.503	0.662<br>0.434-0.890<br>0.354	0.735<br>0.506-0.964<br>0.431	0.646<br>0.415-0.876<br>0.207	0.735<br>0.646-0.864
CNN: RadImageNet DenseNet-121 fine-tuned	0.718<br>0.367-1.000<br>0.399	0.780<br>0.500-1.000<br>0.440	0.738<br>0.496-0.980<br>0.491	0.685<br>0.443-0.926<br>0.431	0.754<br>0.552-0.956<br>0.232	0.738<br>0.685-0.780
CNN: ImageNet ResNet-50 frozen	0.800<br>0.541-1.000<br>0.399	0.818<br>0.604-1.000<br>0.576	0.653<br>0.399-0.908<br>0.244	0.642<br>0.388-0.895<br>0.232	0.673<br>0.453-0.892<br>0.055	0.673<br>0.642-0.818
CNN + ML: RadImageNet ResNet-18 + NB	0.718<br>0.442-0.994<br>0.215	0.727<br>0.438-1.000<br>0.438	0.689<br>0.466-0.912<br>0.164	0.763<br>0.594-0.933<br>0.137	0.700<br>0.494-0.905<br>0.232	0.718<br>0.689-0.763
CNN: ImageNet ResNet-18 fine-tuned	0.741<br>0.452-1.000<br>0.266	0.780<br>0.522-1.000<br>0.251	0.676<br>0.438-0.913<br>0.396	0.613<br>0.370-0.855<br>0.232	0.661<br>0.424-0.897<br>0.366	0.676<br>0.613-0.780
CNN: ImageNet ResNet-50 fine-tuned	0.706<br>0.396-1.000<br>0.482	0.765<br>0.472-1.000<br>0.503	0.698<br>0.445-0.950<br>0.396	0.670<br>0.419-0.921<br>0.358	0.700<br>0.465-0.934<br>0.254	0.700<br>0.670-0.765
CNN + ML: ImageNet ResNet-10t + LR	0.671<br>0.258-1.000<br>0.059	0.886<br>0.661-1.000<br>0.519	0.671<br>0.403-0.940<br>-0.417	0.652<br>0.358-0.947<br>0.473	0.733<br>0.504-0.961<br>0.232	0.671<br>0.652-0.886
CNN: ImageNet ResNet-18 frozen	0.635<br>0.215-1.000<br>0.266	0.727<br>0.409-1.000<br>0.440	0.662<br>0.410-0.914<br>0.254	0.620<br>0.366-0.874<br>0.170	0.643<br>0.392-0.894<br>0.331	0.643<br>0.620-0.727
CNN + ML: ImageNet ResNet-50 + LR	0.671<br>0.385-0.956<br>0.095	0.727<br>0.404-1.000<br>0.000	0.596<br>0.329-0.862<br>-0.013	0.538<br>0.275-0.800<br>0.006	0.544<br>0.332-0.755<br>0.208	0.596<br>0.538-0.727
CNN: RadImageNet ResNet-18 fine-tuned	0.659<br>0.345-0.972<br>0.224	0.705<br>0.445-0.964<br>0.438	0.578<br>0.303-0.852<br>0.417	0.573<br>0.294-0.853<br>0.382	0.538<br>0.265-0.810<br>0.177	0.578<br>0.538-0.705
CNN: ImageNet DenseNet-121 frozen	0.647<br>0.335-0.959<br>0.328	0.735<br>0.466-1.000<br>0.440	0.578<br>0.323-0.833<br>0.093	0.523<br>0.267-0.780<br>0.049	0.592<br>0.328-0.855<br>0.177	0.592<br>0.523-0.735

[a] Metrics reflect final refit holdout-test results. AUC 95% CIs were computed via single-model DeLong variance. MCC was evaluated using a top-25% responders criterion.

[b] All models are shown.

Table S1. Model performance across all development-test splits [a, b]

Model / strategy	Dev. 168 / Test 22<br>5 positives	Dev.162 / Test 28<br>6 positives	Dev. 156 / Test 34<br>9 positives	Dev.150 / Test 40<br>9 positives	Dev. 144 / Test 46<br>9 positives	Across splits
	AUC<br>95% CI<br>MCC	AUC<br>95% CI<br>MCC	AUC<br>95% CI<br>MCC	AUC<br>95% CI<br>MCC	AUC<br>95% CI<br>MCC	Median AUC<br>range

Abbreviations: AUC, area under the receiver-operating-characteristic curve; CI, confidence interval; MCC, Matthews correlation coefficient; NB, naive Bayes; LR, logistic regression.
